# The Childhood Maltreatment Modulates the Impact of Negative Emotional Stimuli on Conflict Resolution

**DOI:** 10.3389/fpsyg.2019.00845

**Published:** 2019-04-26

**Authors:** Xianxin Meng, Shuling Gao, Wenwen Liu, Ling Zhang, Tao Suo, Hong Li

**Affiliations:** ^1^School of Psychology, Fujian Normal University, Fuzhou, China; ^2^Institute of Psychology, Chinese Academy of Sciences, Beijing, China; ^3^School of Sociology and Political Science, Shanghai University, Shanghai, China; ^4^School of Education, Nanyang Normal University, Nanyang, China; ^5^School of Education, Henan University, Kaifeng, China; ^6^College of Psychology and Sociology, Shenzhen University, Shenzhen, China

**Keywords:** executive attention, conflict resolution, childhood maltreatment, arrow Eriksen Flanker Task, emotion

## Abstract

It has been reported that negative emotional stimuli could facilitate conflict resolution. However, it remains unclear about whether and how the impact of negative emotional stimuli on conflict resolution varies depending on childhood maltreatment. To clarify this issue, seventy-nine subjects were required to perform an arrow Eriksen Flanker Task which was presented in the center of emotional pictures. The present study found a significant interaction effect of childhood maltreatment and emotion on executive attention scores in reaction times (RTs) that reflect conflict resolution speed. For subjects in high childhood maltreatment, negative pictures elicited smaller executive attention scores in RTs than neutral and positive pictures, while neutral and positive pictures elicited similar executive attention scores in RTs. By contrast, for subjects in low childhood maltreatment, executive attention scores in RTs were similar across three conditions. These results suggest that the speed of conflict resolution is enhanced in high, instead of low, childhood maltreatment in situations of negative stimuli. This finding extends our understanding of the interaction among emotion, childhood maltreatment and conflict resolution.

## Introduction

Child maltreatment is a global phenomenon affecting the lives of millions of children all over the world (Stoltenborgh et al., 2015; Viola et al., 2016). A recent research reported that across the globe the overall estimated prevalence rates assessing maltreatment ever during childhood were 12.7% for sexual abuse, 22.6% for physical abuse, 36.3% for emotional abuse, 16.3% for physical neglect, and 18.4% for emotional neglect (Stoltenborgh et al., 2015). Existing research has amply demonstrated that exposure to childhood maltreatment is associated with a significantly increased likelihood of multiple forms of psychopathology, including depressive disorder (Nanni et al., 2012; Hovens et al., 2016), anxiety disorder (Bruce et al., 2012; Choi and Sikkema, 2015), bipolar disorder (Pavlova et al., 2016), attention-deficit/hyperactivity disorder (Briscoe-Smith and Hinshaw, 2006) and common psychiatric disorders (Kim et al., 2009; Keyes et al., 2012). It is thus imperative to understand the mechanisms underlying the association between childhood maltreatment and later psychopathology to break the continuity between the two.

It has been proposed that one possible mechanism for the association between childhood maltreatment and later psychopathology is that childhood maltreatment increases sensitization to negative emotional stimuli (Pollak et al., 2001; Sandre et al., 2018). Existing research has demonstrated that childhood maltreatment influences attention bias to negative signals (Pine et al., 2005; Shackman et al., 2007). Using event-related potentials (ERPs) technique, a study conducted by Shackman et al. (2007) found that relative to controls, abused children overattended to task-relevant visual and auditory anger cues, and they also attended more to task-irrelevant auditory anger cues (Pine et al., 2005; Shackman et al., 2007). Furthermore, individuals with childhood maltreatment tend to have difficulties in disengagement of attention from threatening events (Pollak and Tolley-Schell, 2003). Specifically, in a selective attention paradigm using emotional faces as cues, Pollak and Tolley-Schell (2003) found that physically abused children demonstrated delayed disengagement when angry faces served as invalid cues, suggesting the influence of childhood maltreatment on individual’s selective attention to threat-related signals. And the enhanced attention to threat further facilitates both the development and maintenance of emotional disorders (Li et al., 2008; Yuan et al., 2009, 2014, 2015; Meng et al., 2015, 2016).

For the influence of emotion on executive attention control, Easterbrook (1959) influential hypothesis argued that increased emotional arousal of negative stimuli may result in narrowing attention breadth and reducing interference of distracting or irrelevant information (Easterbrook, 1959). In consistence with this view, recent findings observed the facilitation effects of negative stimuli on the processing of conflict resolution (Dennis et al., 2008; Finucane and Power, 2010; Kanske and Kotz, 2010, 2011). For example, when subjects were engaged in identifying the print color of a central target word and ignoring the flanker words above and below the target word, Kanske and Kotz (2011) observed that reaction times (RTs) to incongruent stimuli, in which the target and flanker colors are different, are faster when these stimuli are emotional negative compared to neutral. In addition, when subjects were required to complete a modified version of the Attention Network Test after the presentation of emotional pictures, Finucane and Power (2010) observed that in comparison with neutral stimuli, fear stimuli reduced RTs to a target. Taken together, since individuals with childhood maltreatment experiences tend to develop sensitization to negative stimuli, and negative stimuli could facilitate conflict resolution, it is likely that childhood maltreatment could modulate the impact of negative stimuli on conflict resolution. Specifically, negative stimuli would elicit increased emotion arousal for subjects in high childhood maltreatment, which would narrow their attention and facilitate their conflict resolution. Nevertheless, to date, this hypothesis has not been examined.

To clarify whether and how the impact of negative emotional stimuli on conflict resolution depends on childhood maltreatment, we asked subjects to perform an arrow Flanker Task which was presented in the center of emotional pictures. The arrow Flanker Task (Eriksen and Eriksen, 1974) is a frequently used interference paradigm to investigate conflict resolution (Posner et al., 2007; Finucane and Power, 2010). In the arrow Flanker Task, participants respond to a target arrow presented among strings of flanker arrows, which are either identical with the target (congruent conditions) or different from the target arrows (incongruent conditions). Typically, incongruent conditions elicit slower RTs and more error rates than congruent conditions. Conflict resolution efficiency usually was evaluated by executive attention scores that were calculated by subtracting their responses during congruent conditions from their responses in incongruent conditions (RTs incongruent conditions – RTs congruent conditions; ERs incongruent conditions – ERs congruent conditions) (O’Toole et al., 2011). Specifically, higher scores in ERs indicate reduced conflict resolution accuracy, and higher scores in RTs reflect reduced conflict resolution speed. Given that childhood maltreatment is associated with diminished executive functioning in children, adolescents, and adults (Perez and Widom, 1994; Porter et al., 2005; Minzenberg et al., 2008; Spann et al., 2012). We hypothesize that compared to subjects in low childhood maltreatment, subjects in high childhood maltreatment would show rapid slow conflict resolution in the measurement of executive attention scores in RTs and in ERs. Furthermore, based on previous studies showing individuals with childhood maltreatment experiences tend to develop sensitization to negative stimuli, and negative stimuli could facilitate conflict resolution (Easterbrook, 1959; Dennis et al., 2008; Finucane and Power, 2010; Kanske and Kotz, 2010, 2011), we hypothesize that compared to subjects in low childhood maltreatment, subjects in high childhood maltreatment would show rapid conflict resolution in the measurement of executive attention scores in RTs during the presentation of negative emotional stimuli. As there is no conclusive finding in previous research on the effect of negative emotional stimuli on conflict resolution in the measurement of executive attention scores in ERs, we would not make specific hypothesis for the modulating effect of childhood maltreatment on the impact of emotion on conflict resolution in the measurement of executive attention scores in ERs during the presentation of negative emotional stimuli. This study facilitates the understanding of the mechanisms underlying the association between childhood maltreatment and later psychopathology.

## Materials and Methods

### Subjects

Seventy-nine (43 males, 36 females, mean age: 20.5 years; SD: 2.31) students from Nanyang Normal University were recruited for the experiment. All the subjects were right-handed, and had self-reported normal or corrected-to-normal vision. In addition, subjects reported that they were healthy and free of any reported affective disorders. Each subject provided informed consent prior to the experiment. The experimental procedure was conducted in accordance with guidelines of the 1964 Declaration of Helsinki (World Medical Organization, 1996) and approved by the ethics committee of the School of Psychology in Southwest University.

After consent procedures and before experiment, subjects were required to complete the Childhood Trauma Questionnaire–Short Form (CTQ-SF) scale. The CTQ-SF was used for assessing childhood maltreatment (Bernstein et al., 2003). It is composed of five subscales, including sexual abuse, physical neglect, emotional abuse, physical abuse, and emotional neglect. There are 28 items in total in CTQ-SF and responses are rated on a 5-point scale (ranging from “never true” to “very often true”). According to the childhood maltreatment median score (childhood maltreatment median = 35), we grouped subjects into either a low childhood maltreatment group (*n* = 41, the average score of children maltreatment is 30.27) or a high childhood maltreatment group (*n* = 38, the average score of children maltreatment is 41.87). The low childhood maltreatment group has lower scores in CTQ-SF than the high childhood maltreatment group [*F*(1,78) = 122.92, *p* < 0.001].

### Emotion Induction Stimuli

To avoid the cultural bias in Chinese subjects when the International Affective Picture System was adopted to elicit emotion (Huang and Luo, 2004), the current study selected the emotional pictures from the native Chinese Affective Picture System (CAPS; Bai et al., 2005). In the present study, 112 positive pictures, 112 neutral pictures, and 112 negative pictures were used. The present study included six blocks and each block consisted of 168 trials (grouped into three conditions: positive, neutral, and negative). The size and resolution of all these emotional pictures used in the present study were identical. Additionally, the contrast of the monitor was set to a constant value across subjects.

### Valence and Arousal Assessment

In order to examine the validity of the three pictures sets (negative, neutral, positive), another sample of subjects (*n* = 41; 18 males, 23 females; age ranged from 18 to 25 years; mean age: 21.27) was recruited to rate the valence (negative – neutral – positive) and arousal (low arousal level- high arousal level) of the 336 pictures on a self-reported 9-point rating scale (SAM; Lang et al., 1997). The valence significantly differed amongst negative, neutral, and positive pictures [*F*(2,80) = 265.902, *p* < 0.001]. Positive pictures (6.728) were rated more positive than were neutral pictures (5.369) [*F*(1,40) = 113.701, *p* < 0.001] which, in turn, were rated positive compared with the negative pictures (2.943) [*F*(1,40) = 233.754, *p* < 0.001]. Also, the arousal level significantly differed amongst negative, neutral and positive pictures [*F*(2,80) = 49.069, *p* < 0.001]. Negative pictures (6.44) were rated more arousing relative to positive pictures (5.342) [*F*(1,40) = 32.683, *p* < 0.01] which, again, were rated more arousing than were neutral stimuli (3.646) [*F*(1,40) = 140.221, *p* < 0.001].

### Behavioral Procedures

**FIGURE 1:**
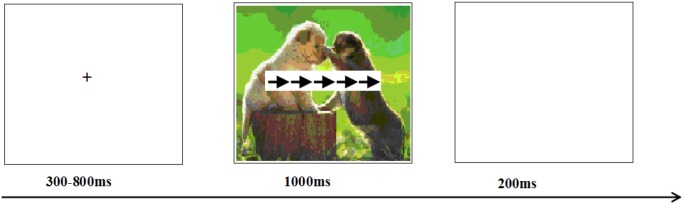
The diagram of our experimental task.

In a dimly room, subjects were seated with 150 cm viewing distance from a computer screen. They were instructed to perform an arrow Eriksen Flanker Task. Each trial started with a 300–800 ms presentation of small black cross on the white computer screen. Then, an arrow Flanker Task which was superimposed on a picture was presented. Subjects were instructed to respond as accurately and quickly as possible. Responses were given with pressing the “F” key on the keyboard if the middle arrow pointed to the left, and pressing the “J” key if the middle arrow pointed to the right. The presentation of the arrow Flanker Task and the emotional picture was simultaneously terminated by a key press or after 1000 ms. Each response was followed by a 200 ms presentation of a blank screen (see Figure 1). Before the formal experiment, all subjects took part in pre-training with 12 practice trials during which they were familiarized with the procedure.

## Results

A repeated measures ANOVA of RTs and ERs was conducted with emotion (positive pictures, neutral pictures, and negative pictures), childhood maltreatment (high, low), and conflict type (congruent, incongruent) as factors. Before determining basic statistical analysis of ERs and RTs, we adopted one-Sample Kolmogorov–Smirnov test to analyze whether the data are suitable for normal distribution. PASW General Linear Model software Version 17 was adopted for statistical analyses (SPSS Inc., 2009). The Greenhouse-Geisser method was used to correct the degrees of freedom of the F-ratio in all these analyses. Simple effects analyses and pair-wise comparisons were conducted using Bonferroni-Holm correction method if significant main effects and interactions were detected.

The normal distribution of ERs significantly deviates. Thus the mean of the ERs was log transformed for the repeated measures ANOVA. We observed a significant main effect of conflict type [*F*(1,77) = 25.985, *p* < 0.001] (see Table 1). Incongruent conditions elicited more false responses than congruent conditions, disregarding of emotion. Furthermore, we observed a significant interaction between conflict type and emotion [*F*(2,154) = 4.074, *p* < 0.05]. The two-way interaction was manifested by the smallest differences between incongruent and congruent conditions during the negative pictures. To better present these results, we computed executive attention scores in ERs by calculating the difference between incongruent and congruent conditions. The repeated measures ANOVA on executive attention scores in ERs showed that negative pictures elicited smaller executive attention scores than neutral [*F*(1,77) = 5.488, *p* < 0.05] and positive pictures [*F*(1,77) = 6.573, *p* < 0.05], whereas neutral and positive pictures elicited similar executive attention scores [*F*(1,77) = 0.182, *p* = 0.671]. No other significant main effects or interaction effects were found for ERs. No other significant main effects or interaction effects were found for ERs.

**Table 1:** During the presentation of negative, neutral and positive pictures, congruent and incongruent conditions elicited average reaction times (in Milliseconds) and error rates for subjects in low and high childhood maltreatment.

		Reaction times		Error rates	
		congruent	incongruent	congruent	incongruent
low childhood maltreatment	negative	499.691(7.012)	536.727(7.242)	2.8% (0.5%)	6.5% (0.9%)
	positive	499.133(6.896)	535.540(6.839)	2.4% (0.4%)	6.8% (1%)
high childhood maltreatment	neutral	496.381(6.622)	530.46(6.964)	2.5% (0.5%)	6.7% (1%)
	negative	496.924(8.067)	526.577(8.332)	3% (0.5%)	5.8% (1%)
	positive	490.956(7.934)	525.550(7.868)	2.8% (0.5%)	6.5% (1.1%)
	neutral	489.237(7.619)	525.066(8.012)	2.4% (0.5%)	6.2% (1.1%)


*Values given in parenthesis were standard errors.*

The normal distribution of RTs does not significantly deviate. The repeated measures ANOVA on RTs showed significant main effects of conflict type [*F*(1,77) = 511.084, *p* < 0.001] and emotion [*F*(2,154) = 11.081, *p* < 0.001]. Incongruent conditions elicited longer RTs than congruent conditions, disregarding of emotion. RTs were longer during negative [*F*(1,77) = 19.107, *p* < 0.001] and positive pictures [*F*(1,77) = 13.698, *p* < 0.001] than during neutral pictures, whereas RTs were similar between negative and positive pictures [*F*(1,77) = 2.652, *p* = 0.101]. More interesting, we observed a three-way interaction amongst emotion, conflict type, and childhood maltreatment [*F*(2,154) = 5.021, *p* < 0.05]. The breakdown of the three-way interaction showed that the interaction between conflict type and emotion was significant in subjects in high [*F*(2,74) = 5.475, *p* < 0.01], instead of low [*F*(2,80) = 1.084, *p* = 0.333], childhood maltreatment. For subjects in high childhood maltreatment, the smallest differences between incongruent and congruent conditions were observed during the negative pictures, whereas for subjects in low childhood maltreatment, similar differences between incongruent and congruent conditions were observed during negative, neutral, and positive pictures. To better present these results, we computed executive attention scores in RTs by calculating the difference between incongruent and congruent conditions. The repeated measures ANOVA on executive attention scores in RTs showed a significant two-way interaction between childhood maltreatment and emotion [*F*(2,154) = 5.021, *p* < 0.05]. The breakdown of the two-way interaction showed a significant emotion effect in subjects in high [*F*(2,74) = 5.475, *p* < 0.01], instead of low [*F*(2,80) = 1.084, *p* = 0.333], childhood maltreatment. For subjects in high childhood maltreatment, negative pictures elicited smaller executive attention scores than neutral [*F*(1,37) = 10.037, *p* < 0.01] and positive pictures [*F*(1,37) = 7.272, *p* < 0.05], whereas neutral and positive pictures elicited similar executive attention scores in RTs [*F*(1,37) = 0.001, *p* = 0.977]. By contrast, for subjects in low childhood maltreatment, executive attention scores in RTs were similar across three conditions (see Table 1). No other significant main effects or interaction effects were found for RTs.

## Discussion

Using an arrow Flanker Task, this study aims to investigate whether the impact of emotion on conflict resolution varies depending on childhood maltreatment. Regardless of emotion, incongruent conditions elicited slower RTs and lower response accuracy than congruent conditions, suggesting that the task used in the present study is effective in inducing attention executive control.

More interesting, our hypothesis that childhood maltreatment modulates the effect of emotion on conflict resolution in the measurement of executive attention scores in RTs was confirmed. Specifically, for subjects in high childhood maltreatment, negative pictures elicited smaller executive attention scores in RTs than positive and neutral pictures. By contrast, for subjects in low childhood maltreatment, similar executive attention scores in RTs were yielded across three emotion conditions. When discussing the effect of negative emotion on cue utilization and the organization of behavior, Easterbrook (1959) argued that increased emotional arousal of negative stimuli may result in narrowing attention breadth and reducing interference of distracting or irrelevant information (Easterbrook, 1959). According to this argument, it is reasonable that negative emotion stimuli elicit increased emotion arousal for subjects in high childhood maltreatment, which narrows their attention to cues in the Flanker Task and thus speeds up their response in conflict resolution in the present study.

For the effect of emotion on conflict resolution accuracy in the measurement of executive attention scores in ERs, our findings showed that negative pictures elicited smaller executive attention scores in ERs than neutral pictures for subjects in both high and low childhood maltreatment. This suggests that subjects in both low and high childhood maltreatment showed the enhancement of the accuracy of conflict resolution in negative emotional stimuli. On the one hand, this finding is consistent with previous research (e.g., Finucane and Power, 2010), suggesting that during negative emotional experience subjects were better able to inhibit irrelevant information resulting in accurate response to a target. On the other hand, this finding indicated that childhood maltreatment did not modulate the effect of emotion on conflict resolution accuracy using the Flanker Task. This maybe because of a “ceiling effect,” that is, the conflict resolution task in the present study is relatively easy for all participants so that it is lack of discrimination validity to produce a significant difference in conflict resolution accuracy.

In contrast with the impact of negative stimuli on conflict resolution, we did not observe the impact of positive stimuli produced on conflict resolution. That is, executive attention scores in ERs and RTs were similar between positive and neutral stimuli in subjects in both high and low childhood maltreatment. According to the motivational intensity theory of affective states (Harmon-Jones et al., 2012, 2013a,b), one possible explanation for these results is that compared to negative pictures, positive and neutral pictures used in this study elicited similar but low level motivational intensity, which did not produce significant influence on narrowing cognitive scope.

## Limitations and Future Directions

A number of important limitations of the present study and future directions should be mentioned. First, negative and positive pictures used in the present study differed not only on valence but also on arousal level. As a result, it is unclear whether the accelerated conflict resolution during the presentation of negative emotional stimuli in subjects in high childhood maltreatment is driven by valence or arousal level. This issue is worthy of further investigation by including positive and negative emotional stimuli of equally low arousal level and equally high arousal level in future studies. Second, the sample size is relatively small. The number of subjects in high childhood maltreatment is 41 and the number of subjects in low childhood maltreatment is 38. The findings of the present study need to be replicated in a larger sample. Third, subjects in this study are healthy. Even the subjects in high childhood maltreatment did not meet criteria for clinical diagnosis of psychiatric population. Hence, the present results need to be replicated in psychiatric population in future studies. Fourth, as there are known modulatory effects of cultural (Butler et al., 2007; Matsumoto et al., 2008; Soto et al., 2011) and age (Meng et al., 2015; Yuan et al., 2015) in the studies of emotional processing, our findings are limited to only Chinese subjects of a small age range. Future studies recruiting larger age range samples of subjects from Chinese and other nations will broaden and increase confidence in the present findings.

## Theoretical and Practical Implications

Despite several limitations, the present study has important theoretical and practical implications for the future studies. Subjects in high childhood maltreatment showed faster response times in conflict resolution in negative emotion situation, which may imply their oversensitivity to negative stimuli in threatening situations. And the oversensitivity to threat further facilitates both the development and maintenance of emotional disorders (Li et al., 2008; Yuan et al., 2009, 2014, 2015; Meng et al., 2015, 2016). The present finding may partly account for why individuals suffering from childhood maltreatment are vulnerable to psychopathology.

Furthermore, if oversensitivity to negative stimuli in threatening situations of subjects in high childhood maltreatment is ultimately shown to contribute to their psychopathology, this observation may provide new therapeutic insights. For instance, new therapies might specifically target underlying abnormalities in sensitivity to negative stimuli as a means of affecting psychopathology.

In summary, the present study demonstrated that the impact of negative emotional stimuli on conflict resolution varied depending on childhood maltreatment. Specifically, subjects in high, instead of low, childhood maltreatment showed an enhanced speed of conflict resolution during the presentation of negative emotional stimuli.

## Ethics Statement

The experimental procedure was in accordance with the ethical principles of the 1964 Declaration of Helsinki (World Medical Organization, 1996). The experimental procedure were approved by the IRB of the School of Psychology in Southwest university.

## Author Contributions

XM and WL conducted the experiments and analyzed the data. XM, WL, SG, and LZ proposed the concept of the measurements. HL helped in the experimental design. XM and HL supervised the project, and conducted the theoretical investigations leading to presented simulations. All authors discussed and contributed to the manuscript.

## Conflict of Interest Statement

The authors declare that the research was conducted in the absence of any commercial or financial relationships that could be construed as a potential conflict of interest.

## References

[B1] Bai L. Ma H. Huang Y. X. (2005). The development of native Chinese affective picture system-A pretest in 46 college students. *Chin. Ment. Health J.* 19:11.

[B2] Bernstein D. P. Stein J. A. Newcomb M. D. Walker E. Pogge D. Ahluvalia T. (2003). Development and validation of a brief screening version of the childhood trauma questionnaire. *Child Abuse Neglect* 27 169–190. 10.1016/S0145-2134(02)00541-0 12615092

[B3] Briscoe-Smith A. M. Hinshaw S. P. (2006). Linkages between child abuse and attention-deficit/hyperactivity disorder in girls: behavioral and social correlates. *Child Abuse Neglect* 30 1239–1255. 10.1016/j.chiabu.2006.04.008 17097140 PMC1934403

[B4] Bruce L. C. Heimberg R. G. Blanco C. Schneier F. R. Liebowitz M. R. (2012). Childhood maltreatment and social anxiety disorder: implications for symptom severity and response to pharmacotherapy. *Depress. Anxiety* 29 131–138. 10.1002/da.20909 22065560 PMC3314083

[B5] Butler E. A. Lee T. L. Gross J. J. (2007). Emotion regulation and culture: are the social consequences of emotion suppression culture-specific? *Emotion* 7 30–48. 10.1037/1528-3542.7.1.30 17352561

[B6] Choi K. W. Sikkema K. J. (2015). Childhood maltreatment and perinatal mood and anxiety disorders: a systematic review. *Trauma Violence Abuse* 17 1–27. 10.1177/1524838015584369 25985988

[B7] Dennis T. A. Chen C. McCandliss B. D. (2008). Threat-related attentional biases: an analysis of three attention networks. *Depress. Anxiety* 25 E1–E10. 10.1002/da.20308 17565734 PMC2662699

[B8] Easterbrook J. A. (1959). The effect of emotion on cue utilization and the organization of behavior. *Psychol. Rev.* 66 183–201. 13658305 10.1037/h0047707

[B9] Eriksen B. A. Eriksen C. W. (1974). Effects of noise letters upon the identification of a target letter in a nonsearch task. *Percept. Psychophys.* 16 143–149.

[B10] Finucane A. M. Power M. J. (2010). The effect of fear on attentional processing in a sample of healthy female. *J. Anxiety Disord.* 24 42–48. 10.1016/j.janxdis.2009.08.005 19729280

[B11] Harmon-Jones E. Gable P. A. Price T. F. (2012). The influence of affective states varying in motivational intensity on cognitive scope. *Front. Integr. Neurosci.* 6:73. 10.3389/fnint.2012.00073 22973207 PMC3437552

[B12] Harmon-Jones E. Gable P. A. Price T. F. (2013a). Does negative affect always narrow and positive affect always broaden the mind? considering the influence of motivational intensity on cognitive scope. *Curr. Dir. Psychol. Sci.* 22 301–307. 10.1177/0963721413481353

[B13] Harmon-Jones E. Harmon-Jones C. Price T. F. (2013b). What is approach motivation? *Emot. Rev.* 5 291–295.

[B14] Hovens J. G. Giltay E. J. van Hemert A. M. Penninx B. W. (2016). Childhood maltreatment and the course of depressive and anxiety disorders: the contribution of personality characteristics. *Depress. Anxiety* 33 27–34. 10.1002/da.22429 26418232

[B15] Huang Y. X. Luo Y. J. (2004). Native assessment of international affective picture system. *Chin. Ment. Health J.* 9 631–634. 10.3758/s13428-014-0535-2 25381023

[B16] Kanske p. Kotz S. A. (2010). Modulation of early conflict processing: N200 responses to emotional words in a flanker task. *Neuropsychologia* 48 3661–3664. 10.1016/j.neuropsychologia.2010.07.021 20654636

[B17] Kanske P. Kotz S. A. (2011). Emotion triggers executive attention: anterior cingulated cortex and amygdala responses to emotional words in a conflict task. *Hum. Brain Mapp.* 32 198–208. 10.1002/hbm.21012 20715084 PMC6870409

[B18] Keyes K. M. Eaton N. R. Krueger R. F. Mclaughlin K. A. Wall M. M. Grant B. F. (2012). Childhood maltreatment and the structure of common psychiatric disorders. *Br. J. Psychiatry* 200 107–115. 10.1192/bjp.bp.111.093062 22157798 PMC3269653

[B19] Kim J. Cicchetti D. Rogosch F. A. Manly J. T. (2009). Child maltreatment and trajectories of personality and behavioral functioning: implications for the development of personality disorder. *Dev. Psychopathol.* 21 889–912. 10.1017/S0954579409000480 19583889 PMC2794554

[B20] Lang P. J. Bradley M. M. Cuthbert B. N. (1997). *International Affective Picture System (IAPS): technical Manual and Affective Ratings.* Gainesville, FL: NIMH Center for the Study of Emotion and Attention.

[B21] Li H. Yuan J. J. Lin C. D. (2008). The neural mechanism underlying the female advantage inidentifying negative emotions: an event-related potential study. *NeuroImage* 40 1921–1929. 10.1016/j.neuroimage.2008.01.033 18343686

[B22] Matsumoto D. Yoo S. H. Nakagawa S. (2008). Culture, emotion regulation, and adjustment. *J. Pers. Soc. Psychol.* 94:925. 10.1037/0022-3514.94.6.925 18505309

[B23] Meng X. Liu W. Zhang L. Li X. Yao B. Ding X. (2016). EEG oscillation evidences of enhanced susceptibility to emotional stimuli during adolescence. *Front. Psychol.* 7:616. 10.3389/fpsyg.2016.00616 27242568 PMC4870281

[B24] Meng X. Yang J. Cai A. Y. Ding X. S. Liu W. Li H. (2015). The neural mechanisms underlying the aging-related enhancement of positive affects: electrophysiological evidences. *Front. Aging Neurosci.* 7:143. 10.3389/fnagi.2015.00143 26300770 PMC4527238

[B25] Minzenberg M. J. Poole J. H. Vinogradov S. (2008). A neurocognitive model of borderline personality disorder: effects of childhood sexual abuse and relationship to adult social attachment disturbance. *Dev. Psychopathol.* 20 341–368. 10.1017/S0954579408000163 18211741

[B26] Nanni V. Uher R. Danese A. (2012). Childhood maltreatment predicts unfavorable course of illness and treatment outcome in depression: a meta-analysis. *Am. J. Psychiat.* 169 141–151. 10.1176/appi.ajp.2011.11020335 22420036

[B27] O’Toole L. J. DeCicco J. M. Hong M. Dennis T. A. (2011). The impact of task-irrelevant emotional stimuli on attention in three domains. *Emotion* 11 1322–1330. 10.1037/a0024369 21707156

[B28] Pavlova B. Perroud N. Cordera P. Uher R. Dayer A. Aubry J. M. (2016). Childhood maltreatment and comorbid anxiety in people with bipolar disorder. *J. Affect. Disord.* 192 22–27. 10.1016/j.jad.2015.12.002 26706828

[B29] Perez C. M. Widom C. S. (1994). Childhood victimization and long-term intellectual and academic outcomes. *Child Abuse Neglect* 18 617–633. 7953902 10.1016/0145-2134(94)90012-4

[B30] Pine D. S. Mogg K. Bradley B. P. Montgomery L. A. Monk C. S. McClure E. (2005). Attention bias to threat in maltreated children: implications for vulnerability to stress-related psychopathology. *Am. J. Psychiat.* 162 291–296. 10.1176/appi.ajp.162.2.291 15677593

[B31] Pollak S. D. Klorman R. Thatcher J. E. Cicchetti D. (2001). P3b reflects maltreated children’s reactions to facial displays of emotion. *Psychophysiology* 38 267–274. 10.1111/1469-8986.3820267 11347872

[B32] Pollak S. D. Tolley-Schell S. A. (2003). Selective attention to facial emotion in physically abused children. *J. Abnorm. Psychol.* 112 323–338. 10.1037/0021-843X.112.3.323 12943012

[B33] Porter C. Lawson J. S. Bigler E. D. (2005). Neurobehavioral sequelae of child sexual abuse. *Child Neuropsychol.* 11 203–220. 16036445 10.1080/092970490911379

[B34] Posner M. I. Rueda M. R. Kanske P. (2007). “Probing the mechanisms of attention,” in *Handbook of Psychophysiology*, eds Cacioppo J. T. Tassinary J. G. Berntson G. G. (Cambridge: Cambridge University Press), 410–432.

[B35] Sandre A. Ethridge P. Kim I. Weinberg A. (2018). Childhood maltreatment is associated with increased neural response to ambiguous threatening facial expressions in adulthood: evidence from the late positive potential. *Cogn. Affect. Behav. Neurosci.* 18 143–154. 10.3758/s13415-017-0559-z 29313252

[B36] Shackman J. E. Shackman A. J. Pollak S. D. (2007). Physical abuse amplifies attention to threat and increases anxiety in children. *Emotion* 7 838–852. 10.1037/1528-3542.7.4.838 18039053

[B37] Soto J. A. Perez C. R. Kim Y.-H. Lee E. A. Minnick M. R. (2011). Is expressive suppression always associated with poorer psychological functioning? A cross-cultural comparison between European Americans and Hong Kong Chinese. *Emotion* 11 1450–1455. 10.1037/a0023340 21707152

[B38] Spann M. N. Mayes L. C. Kalmar J. H. Guiney J. Womer F. Y. Pittman B. (2012). Childhood abuse and neglect and cognitive flexibility in adolescents. *Child Neuropsychol.* 18 182–189. 10.1080/09297049.2011.595400 21942637 PMC3326262

[B39] SPSS Inc. (2009). *Pasw Statistics 17.0.* Chicago: SPSS Inc.

[B40] Stoltenborgh M. Bakermans-Kranenburg M. J. Alink L. R. IJzendoorn M. H. (2015). The prevalence of child maltreatment across the globe: review of a series of meta analyses. *Child Abuse Rev.* 24 37–50. 10.1002/car.2353

[B41] Viola T. W. Salum G. A. Kluwe-Schiavon B. Sanvicente-Vieira B. Levandowski M. L. Grassi-Oliveira R. (2016). The influence of geographical and economic factors in estimates of childhood abuse and neglect using the childhood trauma questionnaire: a worldwide meta-regression analysis. *Child Abuse Neglect* 51 1–11. 10.1016/j.chiabu.2015.11.019 26704298

[B42] World Medical Organization (1996). Declaration of Helsinki (1964). *Br. Med. J.* 313 1448–1449.

[B43] Yuan J. Yang J. Li H. Chen X. Ju E. Meng X. (2015). Enhanced brain susceptibility to negative stimuli in adolescents: ERP evidences. *Front. Behav. Neurosci.* 9:98. 10.3389/fnbeh.2015.00098 25972790 PMC4412063

[B44] Yuan J. J. Chen J. Yang J. M. Ju E. X. Norman G. J. Ding N. X. (2014). Negative mood state enhances the susceptibility to unpleasant events: neural correlates from a music- primed emotion classification task. *PLoS One* 9:e89844. 10.1371/journal.pone.0089844 24587070 PMC3938531

[B45] Yuan J. J. Luo Y. J. Yan J. H. Meng X. X. Yu F. Q. Li H. (2009). Neural correlates of the females’ susceptibility to negative emotions: an insight into gender-related prevalence of affective disturbances. *Hum. Brain Mapp.* 30 3676–3686. 10.1002/hbm.20796 19404991 PMC6870863

